# Urolithiasis-related ureteric intussusception: A rare case report

**DOI:** 10.1016/j.eucr.2023.102626

**Published:** 2023-11-29

**Authors:** Hashim Homaida, Ibrahim A. Khalil, Ahmed Haroon, Jamil Ahmad, Khalid Mahmoud, Hosam Tawfik, Abdelfattah Omran, Abdulqadir Alobaidy, Abdullah A. Al-Ansari

**Affiliations:** Department of Urology, Hamad Medical Corporation, Doha, Qatar

## Abstract

Ureteral intussusception is a rare condition that historically occurs as a complication of ureteral neoplasms or iatrogenic endoscopic procedures. Although the exact mechanism of ureteral intussusception is unclear, most reported cases are due to leading points as malignant or benign masses. Urolithiasis related is rarely reported and can be challenging in stone management as it might decrease the spontaneous stone passage rate. In addition, it will increase the complexity of the endoscopic stone management. We present the second reported case of urolithiasis-related ureteric intussusception presented with urosepsis due to obstructive uropathy, successfully managed by an endourological approach.

## 1 Introduction

Ureteral intussusception is a rarely reported condition; the majority of reported cases are related to different variants of urological benign and malignant tumors, with the least of the cases being secondary to iatrogenic surgical complications. Only one patient reported calculus-related^1^; in ureteral intussusception, usually the proximal ureteral wall telescopes into the distal lumen; theoretically, this condition occurs due to a combination of peristaltic activity, urinary flow, and gravity pulling.^2^, ^3^, ^4^

We present a case of an infected and obstructed kidney that was eventually found to be associated with ureteral intussusception related to urolithiasis.

## 2 Case presentation

**Fig. 1:**
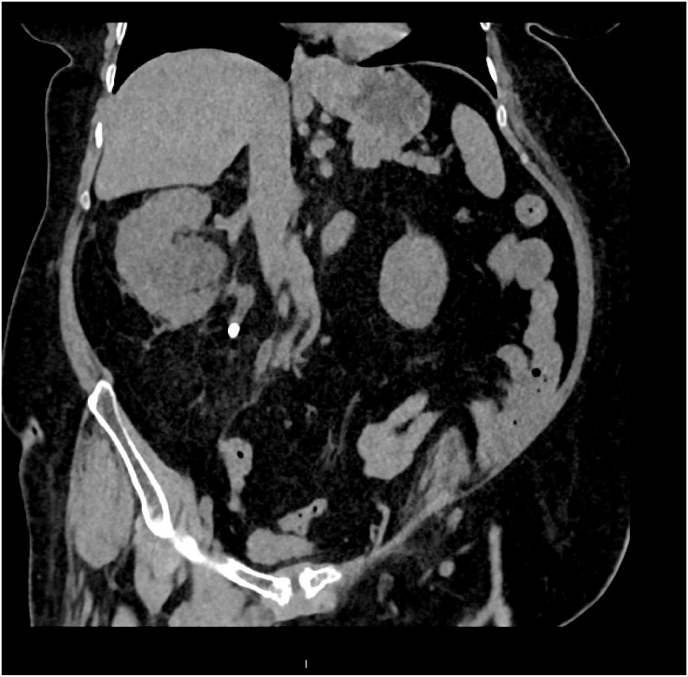
Coronal non-contrast CT scan showing 10 mm obstructive Right proximal ureteric calculus and gross right hydronephrosis.

69 years old lady known hypertensive and diabetic, controlled on medications, with a past medical history of small bilateral renal calculi on conservative treatment presented to our emergency department complaining of severe right flank pain and vomiting that was associated with fever, chills alongside dysuria, and urgency for four days. She was febrile initially, with a temperature of 38.1 c. Workup revealed elevated inflammatory markers with positive nitrates and leukocytes in the urine dipstick. Initial US scan was inconclusive; A non-contrast CT scan confirmed the presence of a 10 × 8 mm calculus in the proximal ureter at the level of L4 (Fig. 1).

The patient was admitted and started on intravenous antibiotics, followed by urgent drainage of the right kidney through an emergency percutaneous nephrostomy tube. The urine culture revealed the growth of *E. coli* ESBL.

After clearing the infection and consulting with the interventional radiology team, the decision was made to intervene retrogradely due to anatomical challenges for antegrade stenting because of abnormal anatomy.

**Fig. 2:**
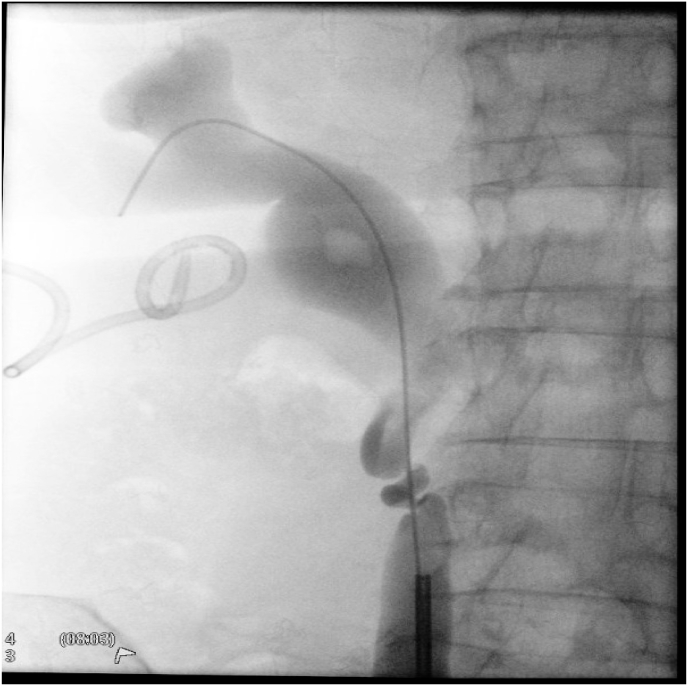
Retrograde Pyelogram showing extremely tortuous upper ureter with a retropulsed stone.

A ureteroscopy was performed. During the procedure, a small stone was seen protruding from the right ureteric orifice, which was removed with forceps, subsequently, a guide wire was advanced up to the right kidney. At that time, a retrograde pyelogram showed a suspicious tortuous upper ureter (Fig. 2).

**Fig. 3:**
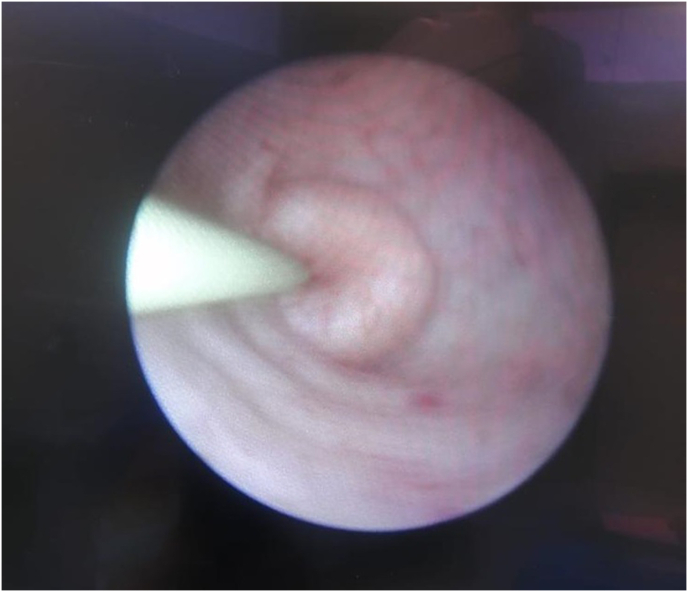
Ureteroscopy showed an intussuscepted ureter with a wire passing into the lumen of the intussusceptum, To the renal pelvis in the ureteric lumen.

Upon advancing the ureteroscope, an upper ureteric intussusception was identified in the ureteric tortuosity below the stone (Fig. 3) (video 1) and was reduced using hydrostatic pressure. A second guide wire was fixed. However, as the stone retropulsed higher, a ureteric access sheath was introduced, followed by a flexible ureteroscope. The stone was fragmented, a stent was left in situ, and the patient was discharged in stable condition.

After one week, the stent was removed during the clinic follow-up, and her subsequent contrasted CT abdomen, after six weeks, revealed only a tiny small renal stone; however, there was no recurrence of the intussusception and no hydronephrosis was observed. During her regular follow-ups, the last US scan showed total clearance of all renal stones and the absence of hydronephrosis.

## 3 Discussion

Ureteral intussusception is a rare condition, as the ureter anatomy prevents ureteric invagination due to multiple variables resulting from the small ratio between ureteral wall thickness, limited range of mobility, and lumen caliber.^5^ Hence, historically, a slow-growing object, commonly a benign mass occupying the ureter, will play an essential role as a focus by pulling down the proximal ureter to the distal dilated ureter by the power of peristaltic activity, gravity, and urine flow.^2^, ^3^, ^4^

The proposed mechanism of urolithiasis-related intussusception is that a non-obstructive ureteric calculus remains in the ureter for an extended period, causing ureteric inflammation. The calculus adheres to the wall of the ureter, and the combination of the antegrade flow of urine, ureteric peristalsis, and gravity pulls the stone in an antegrade direction. As the calculus moves antegrade, it draws on the adherent inflamed ureter, causing intussusception of the ureter, with the intussusceptum containing the calculus and inflamed ureter.^1^ In our case, infection added to the inflammation contributed to intussusception development.

Patients typically present with complaints of hematuria and flank pain. However, it's not universal, as malignant tumors related to ureteric intussusception are often asymptomatic.^5^ Radiological studies helped in diagnosing especially intravenous urography and Contrast CT scan, they demonstrate the primary lesion for intussusception as a filling defect in an enlarged ureteral segment with or without hydronephrosis presenting as a “Stalk – of -corn “.^5^

Management of ureteral intussusception as a complication of chronic ureterolithiasis differs from treatment in the presence of a tumor. As previously reported, cases have been treated with various surgical options, including nephroureterectomy, primary resection and re-anastomosis, boari flap,^1^, ^2^, ^3^, ^4^, ^5^ and, in a unique case, by ileal interposition.^3^ On the other hand, when suspecting a urolithiasis-related intussusception, the ureteroscope is a powerful option for diagnostic and definitive management at the same time, as in our case, the retrograde pyelogram showed suspicious tortuous upper ureter that was confirmed under direct vision, to be a ureteral intussusception, as there was no endoscopic signs of urothelial malignancy, constant hydrostatic pressure and placing two guide wire succeed to straightening the ureter. Additionally, placing a ureteral sheath to clear the stone and avoid using of basket followed by placing double J stent helped in preventing the recurrence of the intussusception.

## Financial disclosure

All the authors declare that they do not have any conflict of interest with the subject of this article.

## CRediT authorship contribution statement

**Hashim Homaida:** Conceptualization, Methodology, Validation, Writing – original draft, Writing - review & editing. **Ibrahim A. Khalil:** Supervision. **Ahmed Haroon:** Writing – original draft. **Jamil Ahmad:** Writing – original draft. **Khalid Mahmoud:** Writing – review & editing. **Hosam Tawfik:** Supervision. **Abdelfattah Omran:** Supervision. **Abdulqadir Alobaidy:** Supervision. **Abdullah A. Al-Ansari:** Supervision.

## Abbreviations

US: ultrasound
CT: computed tomography
mm: millimeter
ESBL: Extended-spectrum beta-lactamases
E.coli: Escherichia coli

## Appendix A Supplementary data

Video 1: Endscopic view of an intussuscepted ureteric segment blow the obstructive stone site.
